# Tunneled Peritoneal Catheter Placement in Palliation of Malignant Ascites: A Study with Two Different Types of Catheters

**DOI:** 10.1155/2019/4132396

**Published:** 2019-05-30

**Authors:** Ahmet Kaya, Omer F. Nas, Cuneyt Erdogan

**Affiliations:** ^1^Radiology Clinic, Bilecik State Hospital, Bilecik, Turkey; ^2^Department of Radiology, Uludag University Faculty of Medicine, Bursa, Turkey; ^3^Radiology Clinic, Medicana Bursa Hospital, Bursa, Turkey

## Abstract

**Objective(s):**

Malignant ascites (MA) is abnormal accumulation of fluid in the peritoneal cavity and has negative effects on the quality of life. The purpose of this retrospective study is to explore feasibility, safety and efficacy of tunneled peritoneal catheter placement using both peritoneal dialysis and hemodialysis catheters in the palliation of MA.

**Methods:**

Between October 2013-June 2016, thirty patients with resistent MA underwent tunneled peritoneal catheterisation in our interventional radiology department. Tunneled peritoneal catheter (TPC) was placed in 22 (n=22/30; %73) patients, tunneled hemodialysis catheter (THC) was placed in 8 patients (n=8/30; %27). Routine visits were scheduled for months 1, 3, 6, 9, and 12 of the catheterization, and the records were evaluated retrospectively.

**Results:**

The overall duration of catheterization varied from 2 to 334 days (mean 66.4 ± 68.5, median: 57 days). Catheters remained intact in 29 patients (96.7%) until the endpoint. There was one (3.3%) malfunctioning catheter among both groups. Overall, four patients developed infection, which were classified into major (n=2/30, %6.7) and minor (n=2/30, %6.7) complications according to SIR criteria.

**Conclusion:**

Tunneled peritoneal catheterization using both TPCs and THCs provided a safe method with relatively high patency, and low infection and systemic complication rates in the palliation of MA.

## 1. Introduction

Malignant ascites (MA) is the abnormal accumulation of fluid in the peritoneal cavity. Peritoneal membrane involvement in some abdominal and extra-abdominal malignancies, changes in vascular permeability, lymphatic obstruction, hepatic congestion due to diffuse hepatic metastases, and exudative fluid secretion from tumor tissue play a role in the pathophysiology of MA [1].

Increased intra-abdominal pressure induced by MA may cause abdominal pain, nausea, loss of appetite, dyspnea, reduced mobility, and cosmetic and psychological problems, including depression, all of which negatively affect quality of life [2]. Treatments with tunneled peritoneal catheter insertion, peritoneovenous shunts, large-volume paracentesis, diuretics, intracavitary chemotherapy, immunotherapy, radioisotope treatment, and laparoscopic hyperthermic intraperitoneal chemotherapy (HIPEC) are the main modalities adopted for the management of MA [3].

Studies in the relevant literature have noted the safety and efficacy of tunneled peritoneal catheter placement using only certain types of catheters. The purpose of our retrospective study is to explore the safety and efficacy of tunneled peritoneal catheter placement using both peritoneal dialysis and hemodialysis catheters in the palliation of MA. To the best of our knowledge there are no studies in the literature using tunneled hemodialysis catheters in the palliation of MA.

## 2. Materials and Methods

Between October 2013 and June 2016, 30 patients with MA who required large-volume paracentesis at least once every week for 1 month underwent tunneled peritoneal catheterization at our interventional radiology department. 15F 62-cm-long double-cuffed peritoneal dialysis catheters (Argyle Peritoneal Dialysis Catheter, Covidien, LLC, MA, USA) were inserted in 22 patients (n=22/30; 73%), and 14.5 F 32-cm-long split-tip hemodialysis catheters (HemoSplit Long-Term Hemodialysis Catheter, Bard, Inc., USA) were inserted in 8 patients (n=8/30; 27%).

Patients' primary malignancies are summarized in Table 1. In one patient, who had both MA and bone metastasis, a bone biopsy indicated a metastatic adenocarcinoma; however, the patient died before the primary site could be identified. Of the 30 patients, 13 (n=13/30; 43.3%) had liver metastases, and 13 (n=13/30; 43.3%) had peritonitis carcinomatosa.

The exclusion criteria consisted of confirmed ascites infection, loculated ascites, presence of thick fibrotic septa, INR above 1.5, and platelet levels below 50,000. The presence of thick fibrotic septa was determined through the use of ultrasound by employing the following two criteria: a septal diameter greater than 3 mm and unresponsiveness of septa to coarse vibration induced by the ultrasound probe. Catheterization was not performed on patients with thick and rigid septa.

After obtaining patients' informed consent, catheterization was performed under sonographic and fluoroscopic guidance at our interventional radiology suite. The entire procedure was performed under local anesthesia, and no need arose for general anesthesia or conscious sedation. After completion of the procedure, all patients were put on oral penicillin (1000 mg of amoxicillin/clavulanate potassium) for 1 week, with the exception of patients already on antibiotic treatment. This retrospective study was approved by the local ethics committee of our institution.

### 2.1. Operative Technique

**Figure 1:**
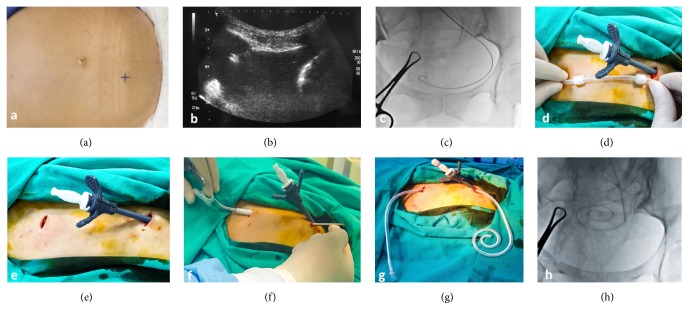
*Operative technique*. (a) Marking the entry site. (b) Accessing the intraperitoneal space with an 18G Chiba needle. (c) Fluoroscopic image showing advancement of the stiff guidewire to the most dependent place in pelvis. (d) Placing the peel-away sheath to the intraperitoneal cavity and determining the site of second incision line. (e) Making the second incision superolateral to the first incision. (f, g) Advancing the tunneling device through the tract with the catheter attached to its backside. (h) Fluoroscopic image showing placement of the catheter to the most dependent part of pelvis.

Prior to the procedure, the right lower quadrant was examined with ultrasonography. The lateral or medial of the inferior epigastric arteries was marked as the entry site (Figure 1(a)).

Under standard surgical sterilization and appropriate draping, a 1-cm incision was made. The intraperitoneal space was accessed with an 18G Chiba needle under sonographic guidance (Figure 1(b)). A sample of about 20 mL of ascites fluid was collected for further laboratory examination.

Under fluoroscopic guidance, a 0.035-inch stiff guidewire (Amplatz Super Stiff, Boston Scientific, USA) was advanced to the most dependent part of the pelvis (Figure 1(c)). After serial dilatation of the tract, a peel-away sheath was placed in the peritoneal cavity over the stiff guidewire (Figure 1(d)). A second incision was made superolateral to the first incision, ensuring that the catheter cuff (both cuffs in a peritoneal catheter group) remained in the tunnel tract delineated by the incision lines (1 cm from incision lines) (Figures 1(d) and 1(e)).

The local anesthetic was injected into the tunnel tract. The tunneling device was advanced through the tract with the peritoneal catheter attached to its backside (Figures 1(f) and 1(g)). The catheter was placed in the most dependent part of the pelvic cavity (Figure 1(h)) through the peel-away sheath, and then the peel-away sheath was removed. The skin was closed with primary suturing.

Immediately after the completion of the procedure, 3 L of ascites was drained to relieve abdominal tension for better wound healing and to prevent ascites leakage. Patients and their caregivers were instructed in catheter care and fluid drainage. Patients were evaluated during the first week of catheterization for early complications, including hematoma, catheter dislodgement, ascites leakage, and wound healing problems. Routine visits were scheduled for Months 1, 3, 6, 9, and 12 of catheterization.

Complications were classified into major and minor groups according to the Clinical Practice Guidelines published by the Society of Interventional Radiology [4]. Study endpoint was determined as the following: end of the study, death, or removal of the catheter for reasons other than occlusion.

### 2.2. Statistical Analysis

The SPSS 22.0 software program was used to analyze the data. The distribution of the variables was measured by using the Kolmogorov–Smirnov test. The Mann–Whitney U test was used to analyze quantitative data. The chi-square test was performed to analyze qualitative data, and Fisher's exact test was used when the test conditions did not meet the assumptions. A p value of less than 0.05 was accepted as statistically significant.

## 3. Results

Technical success rate was 100% for both types of catheters. There were 11 male and 19 female patients. The patients' ages were 25–73 years (mean 54.4 ± 11.7). Demographic features are stated in Table 1. There was no significant difference in patient numbers and demographic features between the tunneled hemodialysis catheter (THC) and tunneled peritoneal catheter (TPC) groups (Table 2).

The overall duration of catheterization varied from 2 to 334 days (mean 66.4 ± 68.5, median 57). The catheter duration in the THC group ranged from 7 to 334 days (mean 84.9 ± 103.9, median 57), and it ranged from 2 to 195 days (mean 59.6 ± 51.9, median 52) in the TPC group (Table 2). There was no significant difference between catheter survival times for the THC and TPC groups (p = 0.79). Total amount of ascites drained from each catheter until the study endpoint ranged from 2 to 190 L (mean 59.27±50.8, median 52) in the TPC group, and it ranged from 2 to 340 L (mean 85.12±106.7, median 60) in the THC group (Table 2). There was no significant difference in drained ascites amount between the two groups (p = 0.85). Of the 30 patients, 27 died of underlying diseases. None of the patients developed a procedure related complication.

In the THC group, one patient (n=1/8; 12.5%) had a catheter malfunction due to the existence of septa, which then resulted in the removal of the catheter on Day 7 of catheterization. There were no other complications related to THC group. One patient with breast cancer received intraperitoneal cisplatin therapy via the catheter.

In the TPC group, major complications occurred in two patients (n=2/22; 9%) due to bacterial peritonitis. One patient, who developed bacterial peritonitis on Day 37 of the procedure, died of pulmonary embolism 1 week later. The other patient developed bacterial peritonitis on Day 48 of catheterization. This patient refused treatment and chose to leave the hospital. On Day 62 of catheterization, the patient died. It is unknown whether the patient died due to peritonitis or some other unrelated cause. Besides the major complications, two patients (n=2/22; 9%) developed minor complications in the form of tunnel tract infection and cellulitis around the tunnel tract. One patient developed a tract infection on Day 67 of catheterization, and he was not responsive to oral antibiotics. Development of abdominal pain and fever raised suspicions of peritonitis, which led to the removal of the catheter. However, the ascites fluid sample did not demonstrate any findings of peritonitis. The patient with cellulitis responded to oral antibiotics, and cellulitis soon disappeared. Although major and minor complication rates were higher in the TPC group, this was found to be statistically insignificant when compared to the THC group, which had no such complications (Table 2).

## 4. Discussion

Our study suggests that tunneled peritoneal catheter placement in palliation of MA, regardless of the catheter type we used, is feasible with a technical success rate of 100% and no immediate complications. Catheters remained in situ until the time of death in 29 patients (96.7%). There was only one (3.3%) malfunctioning catheter among both groups, which was eventually removed. Overall, four patients developed infection during the follow-up period, which were classified into major and minor complications according to SIR criteria. Our study also revealed that there were no significant differences in catheter survival times and complication rates between the THC and TPC groups (Table 2).

Palliative care is valuable in alleviating symptoms of MA, such as distension, respiratory distress, loss of appetite, and reduced mobility, that affect quality of life [2]. The peritoneal dialysis catheter was first used for palliation of MA by interventional radiologists in 2001 [5]. Recent studies in this field mainly pointed to the PleurX catheter, Tenckhoff catheter, and peritoneal dialysis catheter [5–16]. There is no consensus about which type of catheter is most suitable in palliation of MA [5, 13–15]. To the best of our knowledge this study is the first in which tunneled hemodialysis catheter is used for this purpose.

The technique used in this study resembled that of O'Neill et al. [5], who were the first to use the technique; however, we chose to skip introducing a 5 F angiographic catheter through the peritoneal catheter to create a coaxial system. Considering the fact that the success rate of our technique was 100%, the technique in the study of O'Neill et al. [5] can be regarded as both a loss of time and an unnecessary use of materials. Our technique further differed from that of O'Neill et al. [5] in terms of the direction of the tunnel, as we created the tunnel obliquely along the superoinferior axis instead of the lateromedial axis. This direction was chosen to make the catheter place in the pelvis and to avoid ascites leak and accumulation in the tunnel tract. In this respect, our study was similar to that of Akinci et al. [14].

As tunneled catheters are designed for long-term use, it is important that they remain functional for the target duration of use without creating the need for repeated revisions. In the literature, 65–96% of catheters have been reported to remain patent and functional until the endpoint [5–12, 14–16]. Catheter occlusion/impaired drainage frequencies reported in the literature varied from 2.5% to 11% [8–10, 14, 15]. Various techniques were employed in these studies to maintain the lumen patency, including manipulation with guidewire or bronchoscopic brush to tissue plasminogen activator (tPA) infusion. In our study, overall one catheter (%3.3) had to be removed because of drainage-related problems. In this respect, we are convinced that an initial examination for detection of large peritoneal masses or loculated ascites, identification of the most suitable peritoneal compartment, and characterization of rigid septa are important. Our study also revealed that there was no significant difference in catheter survival times between the THC and TPC groups (Table 2).

Catheter infection is a significant problem for all types of catheters. Creating a tunnel underneath the skin and triggering fibrosis with the catheter cuffs play a significant role in avoiding infections. However, despite all efforts to avoid a potential infection, localized skin infections, peritonitis, and some infrequent systemic infections, including sepsis, still pose significant problems among patients. The reported frequency of skin infections varied from 1.5% to 10% in the literature [10, 11, 14–16]. In our study, overall two patients (n=2/30; 6.7%) developed localized infections. The patient with the tunnel tract infection admitted that he had started to neglect his catheter care and hygiene. This points to the importance of providing patient education regarding catheter care and hygiene during routine visits.

The reported frequency of peritonitis, which is a more serious complication compared to localized infections, varied from 1% to 20% in previous studies [5, 6, 9, 10, 14]. In our study, overall two patients (n=2/30; 6.7%) developed bacterial peritonitis. Poor immunity associated with the fact that both patients stayed in the hospital due to poor overall health may have led to the development of peritonitis.

The reported frequency of ascites leak varied from 2 to 21% in previous studies [5, 8–11, 15]. No ascites leak was reported in the studies of O'Neill et al. [5] and Akinci et al. [14], both of which used double-cuffed peritoneal dialysis catheters. In our study, neither the patients with double-cuffed peritoneal dialysis catheters nor those with single-cuffed hemodialysis catheters experienced such complications. The use of double-cuffed catheters contributed to the maturation of the tunnel and helped prevent ascites leakage as well as infections. However, it is not only the number of cuffs but also the tunneling direction that is relevant to ascites infiltration. In the study by Courtney et al. [9], which had an ascites leakage rate of 21%, the leakage was completely resolved through a change in the direction of the tunnel tract to the superoinferior direction.

Dislodgement of tunneled catheters is less likely when compared to nontunneled pigtail catheters. Fibrosis in the tunnel tract acts as a shield that keeps the catheter in place. Nevertheless, studies have reported a dislodgment frequency of 1–14% in patients with tunneled catheters [8–11, 14, 16]. No such complication was observed in our study. In addition to using cuffed catheters, this may also have been due to the craniocaudal tunneling direction and strict patient education about catheter care.

In our study, quality of life was not assessed through the use of a structured questionnaire based upon objective criteria. Instead, patients' self-reported levels of satisfaction were noted. Unlike other studies on tunneled peritoneal catheterization, Courtney et al. [9] employed a questionnaire to assess the quality of life with questions about physical condition, mood, satisfaction with social life, and overall quality of life.

As for the cost-effectivity of the catheters, companies generally avoid giving a fixed price because both marketing conditions of retail sale change in each case and catheter prices are indexed to currency exchange rates. The THC catheter came with access needle, guidewire, peel-away sheath, and dilators as a whole set, and the approximate prices ranged from $ 100 to 150. As for the TPC, beside the catheter, some extra types of equipment also were needed to be bought for the procedure like access needle, stiff guidewire, dilators, and peel-away sheath. Thus, the approximate price of TPC for each patient ranged to over $ 150. In addition, because the TPC was not as common as the THC in our district, the waiting time was much longer for each patient. Therefore, we had to use THC in some of our patients which was the first in the relevant literature. By this, we also had the opportunity to reveal the safety and efficacy of THC in the palliation of MA, although the catheter is primarily designed for long-term hemodialysis.

The main limitation of our study was its retrospective nature and relatively small number of patients. In addition, the study did not employ a structured questionnaire based upon objective criteria. Some of the patients bought the equipment on their own; therefore we do not have the precise catheter prices for all cases which would allow us to reveal an objective quantitative analysis of the cost-effectivity in each group. Prospective studies involving more patients and structured follow-up questionnaires based on objective criteria are required to establish the safety and efficacy of tunneled peritoneal catheterization in palliation of MA.

The results of this study revealed that tunneled peritoneal catheterization using both TPCs and THCs provided an efficacious and safe method with relatively high patency rates and low infection and systemic complication rates in the palliation of MA.

## Figures and Tables

**Table 1:** Primary origins of malignancy.

Primary malignancy	n	n/N, %
Gastric cancer	10	33.4%
Breast cancer	4	13.4%
Colorectal cancer	3	10.0%
Ovarian cancer	3	10.0%
Pancreas cancer	3	10.0%
Hepatocellular cancer	2	6.7%
Cholangiocellular cancer	1	3.3%
Jejunum cancer	1	3.3%
Bladder cancer	1	3.3%
Mesothelioma	1	3.3%
Unknown origin	1	3.3%

**Table 2:** Patients' demographic features and results.

Demographics	TPC	THC	p
Age (mean±SD)	25-73 (54.68±11.2)	27-67 (53.75±13.95)	0.78^m^
Male (%)	9 (%41)	2 (%25)	0.42^X^2^^
Female (%)	13 (%59)	6 (%75)	

Results			

Catheter days	2-195 (59.64±51.92; Median: 52)	7-334 (84.9±103.9; Median: 57)	0.80^m^
Drained amount of ascites (mean±SD)	2-190 L (59.27±50.8; Median: 52)	2-340 L (85.12±106.7; Median: 60)	0.85^m^
Occlusion	0	1 (%12)	0.27^X^2^^
Major complications	2 (%9)	0	1.00^X^2^^
Minor complications	2 (%9)	0	1.00^X^2^^

_ _^m^Mann-Whitney u test/ _ _^X^2^^Chi-square test.

SD: standard deviation/n: number.

THC: tunnelled haemodialysis catheter.

TPC: tunnelled peritoneal catheter.

L: litres.

## Data Availability

Previously reported journal articles data were used to support this study and are are cited at relevant places within the text.
